# Precision of mangrove sediment blue carbon estimates and the role of coring and data analysis methods

**DOI:** 10.1002/ece3.9655

**Published:** 2022-12-25

**Authors:** Paula Sternberg‐Rodríguez, Paula Ezcurra, Matthew T. Costa, Octavio Aburto‐Oropeza, Exequiel Ezcurra

**Affiliations:** ^1^ Centro de Investigaciones Biológicas del Noroeste AC La Paz Baja California Sur Mexico; ^2^ Climate Science Alliance San Diego California USA; ^3^ Scripps Institution of Oceanography University of California San Diego California USA; ^4^ Department of Botany & Plant Sciences University of California Riverside California USA

**Keywords:** blue carbon, bulk density, mangrove, precision, sediment core

## Abstract

Carbon accumulation in coastal wetlands is normally assessed by extracting a sediment core and estimating its carbon content and bulk density. Because carbon content and bulk density are functionally related, the latter can be estimated gravimetrically from a section of the core or, alternatively, from the carbon content in the sample using the mixing model equation from soil science. Using sediment samples from La Paz Bay, Mexico, we analyzed the effect that the choice of corer and the method used to estimate bulk density could have on the final estimates of carbon storage in the sediments. We validated the results using a larger dataset of tropical mangroves, and then by Monte Carlo simulation. The choice of corer did not have sizable influence on the final estimates of carbon density. The main factor in selecting a corer is the operational difficulties that each corer may have in different types of sediments. Because of the multiplication of errors in a product of two variables subject to random sampling error, when using gravimetric estimates of bulk density, the dispersion of the data points in the estimation of total carbon density rises rapidly as the amount of carbon in the sediment increases. In contrast, the estimation of total carbon density using only the carbon fraction as a predictor is very precise, especially in sediments rich in organic matter. This method, however, depends critically on the accurate estimation of the two parameters of the mixing model: the bulk density of pure peat and the bulk density of pure mineral sediment. The estimation of carbon densities in peaty sediments can be very imprecise when using gravimetric bulk densities. Estimating carbon density in peaty sediments using only the estimate of organic fraction can be much more precise, provided the model parameters are estimated with accuracy. These results open the door for simplified and precise estimates of carbon dynamics in mangroves and coastal wetlands.

## Graphical abstract

Carbon accumulation in coastal wetlands is assessed by extracting a sediment core and estimating its carbon content and bulk density. Because carbon content and bulk density are functionally related, the latter can be estimated from the carbon content in the sample. Estimating carbon density in peaty sediments using only the organic fraction can be very precise, provided the model parameters are estimated accurately.

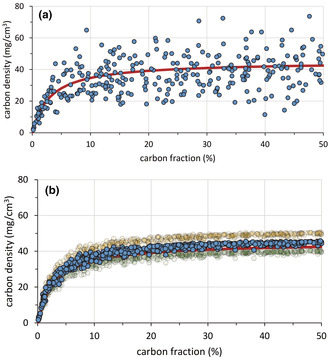

## 1 INTRODUCTION

Due to the anoxic and salty conditions in mangrove substrates, root remnants and litterfall accumulate in the sediments making them one of the most carbon‐rich ecosystems on Earth (Adame et al., 2021; Donato et al., 2011; McKee et al., 2007). Because of the importance of atmospheric carbon sequestration by mangrove ecosystems and its long‐term trapping in the flooded substrate either as peat or as amorphous organic matter, many studies have devoted efforts to estimate the amount of carbon trapped in mangrove sediments as a key input in the calculation of their ecosystem services and their relevance for the growing market for carbon emissions mitigation. This research on mangrove ecosystem services is of high priority given the rapid historical (Valiela et al., 2001) and ongoing (Goldberg et al., 2020) rates of mangrove deforestation, at the same time, climate change, biodiversity loss, and other sustainability crises place immense pressures on coastal communities (Bindoff et al., 2019).

Most studies assessing carbon accumulation in mangrove sediments throughout the tropics follow similar methodologies (Kauffman & Donato, 2012): (a) First, a core is extracted using a sediment corer, which may differ among studies in corer type and depth cored. (b) Then, a segment of the core is cut for analysis, and its volume is estimated by multiplying the length of the segment by the cross‐sectional area of the core. (c) The segment is dried in a low‐heat oven (60–80°C) until constant weight, and the bulk density of the sediment is calculated by dividing the dry mass by the volume. (d) Finally, a subsample is weighed out and analyzed in the lab for its carbon content. Usual methods are loss‐on‐ignition, which estimates total organic matter, or mass proportion of elemental carbon estimated with an elemental analyzer (after HCl treatment to remove carbonate). Total organic matter can be converted into carbon fraction dividing by a conversion factor that may vary slightly from site to site but usually ranges between 2.0 and 2.2 (Pribyl, 2010). The carbon density, i.e., the mass of carbon in a given volume of the sediment, is then obtained by multiplying the bulk density (g cm^−3^) by the proportion of carbon or carbon fraction.

The different coring diameter and sediment‐cutting procedures of each corer in the field could potentially compact, exclude, or otherwise disturb the sediment differently, resulting in altered estimates of bulk density, a critically important element in the estimation of total carbon content. Some studies use standard soil probes (Ezcurra et al., 2016), which have a 17 mm internal bit diameter (when fitted with a tip‐bit for swampy sediments). Others use a larger, 6‐cm‐diameter, open‐faced corer designed for swampy substrates (Donato et al., 2011). Other researchers use the Russian peat corer, which takes semi‐cylindrical cores 5 cm in diameter (McKee et al., 2007) while, finally, some have used a 10‐cm‐diameter core if they need a large sample for other analyses in addition to C content (Smoak et al., 2013). The soil probe and the open‐faced corer cut through the sediment, roots, and peat as they are driven down into the substrate, while the Russian peat corer is driven down empty to the desired depth and closed by rotating the corer to enclose a sample. Carbon accumulation methodology has become standard and is used in almost all sediment blue carbon studies, but little is known about the influence of the type of corer used on the final results.

Additionally, the bulk density of mangrove sediments is not independent of their organic matter content (Callaway et al., 2012; Holmquist et al., 2018; Morris et al., 2016). A sediment with no organic matter will have the bulk density of the mineral matrix, usually a value close to 1.6–2.0 g cm^−3^ in coastal substrates (Holmquist et al., 2018; Morris et al., 2016). Similarly, a sediment formed by pure organic matter will have the bulk density of pure peat, a value normally close to 0.09 g cm^−3^. Any sediment containing a mixture of mineral particles and peat will have a bulk density between those extreme values. It seems possible, then, that the bulk density of a coastal sediment core could be approximately estimated directly from the proportion of carbon or organic matter in the core, eliminating the need to estimate bulk density from the volume and mass of the segment. The question arises, what would be the appropriate model to estimate bulk density from carbon content, and how precise would that procedure be compared to the bulk densities estimated gravimetrically from core segments?

In this study, we address the question above by (a) comparing sediment bulk densities obtained from three different corers to evaluate how much they differ, and (b) comparing carbon estimates obtained from bulk densities calculated from the conventional gravimetric method against carbon estimates obtained from bulk densities that were predicted from the organic matter content of the sediment.

## 2 METHODS

### 2.1 Equipment

**FIGURE 1:**
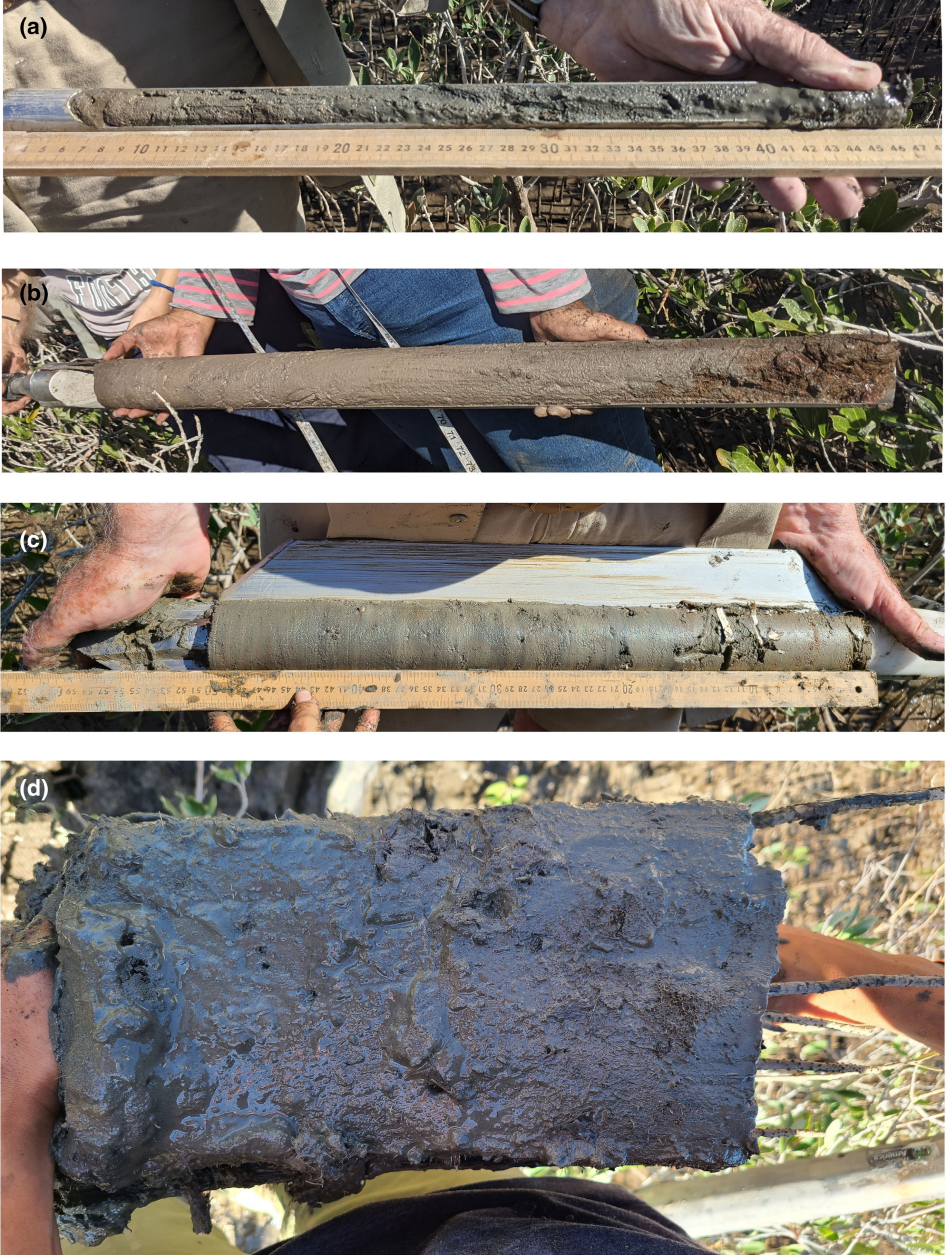
The four bulk density estimation methods: (a) standard soil probe, (b) open‐faced peat corer, (c) Russian peat corer, and (d) rectangular spade to dig out undisturbed aggregates of sediment.

Three corers were used: (a) a standard soil probe (Oakfield Apparatus), (b) a custom‐made open‐faced peat corer (following Kauffman & Donato, 2012), and (c) a Russian peat corer (Belokopytov & Beresnevich, 1955; Jowsey, 1966). Finally, a rectangular spade was used to dig out large aggregates of undisturbed sediments to estimate the true bulk density of sediments at the site (Figure 1).

The soil probe (Figure 1a) has a 30.48 cm sediment‐coring tube with 19 mm inner diameter, a detachable sharp tip, and 30 cm extension rods. We used a wet‐soil tip with 17 mm coring diameter to allow the sample cores to enter easily into the 19 mm tube and to retain the core on extraction. The tube has a cut‐out in the front to allow for sediment sampling, visual inspection, and cleaning.

The open‐faced corer (Figure 1b) is a stainless‐steel single chamber with an inner diameter of 60 mm. The relatively large diameter is intended to reduce vertical compaction of the core by reducing the percentage of the sampled area in close contact with the corer walls. The core chamber is 101.6 cm in length and has extension rods that allow the corer to go deeper when necessary.

The Russian peat corer (Figure 1c) is operated by inserting the corer to the depth interval to be sampled and rotating corer so that the cutting edge moves horizontally around a column of sediment adjacent to the corer while a vertical fin remains anchored in place, sealing the sample in the core chamber without vertical compaction. The model used in this study has a core chamber 50 cm long and samples a cross‐sectional area of 8.81 cm^2^ (Appendix S1).

### 2.2 Field sampling procedure

**FIGURE 2:**
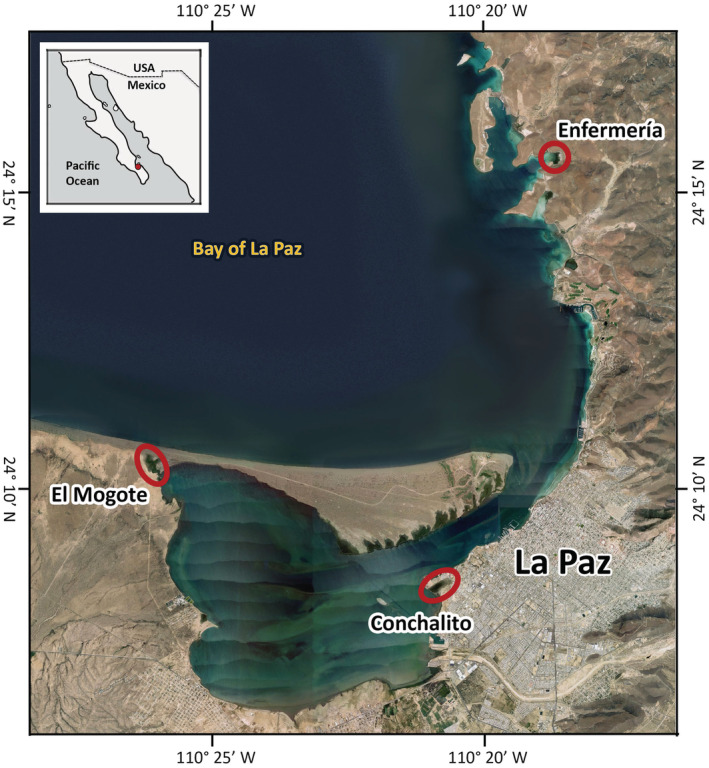
Mangrove field sites in La Paz Bay, Baja California Sur, Mexico: El Conchalito, El Mogote, and Enfermería. Each mangrove forest was sampled at different points at each site to include different sediment types (see text for details; background satellite image courtesy of Google Earth©, date 4/3/2018).

All samples were taken in La Paz Bay, Baja California Sur, in three different mangrove forest locations: El Conchalito (C), El Mogote (M), and Enfermería (X). In an effort to represent different sediment types, including mud, peat, clay, and sand, each mangrove forest was sampled in different sampling sites within each location. El Conchalito was sampled at three sites: C1 (24°08.309′, −110°20.863′), C2 (24°08.464′, −110°20.819′), and C3 (24°08.453′, −110°20.814′). El Mogote was sampled at sites M1 (24°10.300′, −110°26.000′) and M2 (24°10.336′, −110°26.175′). Enfermería was sampled at sites X1 (24°15.691′, −110°18.637′) and X2 (24°15.635′, −110°18.628′; Figure 2).

Within each site, we extracted sediment cores to a maximum depth of 1 m below the substrate surface. From these cores, we cut out segments of known volume at different depths. Each core segment formed our individual sampling unit. Core samples were taken at two or three different depths: One sample was taken at approximately 15 cm belowground, another one at a depth of 40 cm, and, when corer penetration allowed, a third sample was taken in some sites at a depth that fell between 75 and 95 cm. All three corers were used at each sampling site, extracting cores as close to each other as field conditions permitted and avoiding coring on trampled sediments. In some sites, we also extracted a spade sample of undisturbed sediment. For spade samples, only one sample was taken per sampling site at a depth of 15 cm if the upper layer of sediment was not waterlogged and a large intact aggregate could be extracted. Digging out intact aggregates from deeper layers was not possible in the mangroves' waterlogged substrate.

For the soil probe, all samples taken had a vertical extent of 5 cm (10–15 or 35–40 cm below the sediment surface), so each sample had a volume of 11.35 cm^3^. For the open‐faced corer, all samples taken had a vertical extent of 3 cm and a depth range 12–15 and 37–40 cm below the sediment surface, so each sample was a half cylinder, with a volume of 42.41 cm^3^. For the Russian peat corer, all samples had a vertical extent of 3 cm and depth ranges of 12–15 or 37–40 cm, so each semi‐cylindrical sample had a volume of 26.46 cm^3^. In the case of the spade, a rectangular prism with approximate dimensions 16.5 × 16.5 × 30 cm was carefully cut out of the ground in order to cause minimal disturbance. Once the large block of sediment was retrieved, a small 3 × 3 × 3 cm cube was carefully sliced with a sharp knife to cause the lowest disturbance possible. All spade samples were in the depth range 12–15 cm and had a volume of 27 cm^3^.

### 2.3 Experimental design

In each core sample, we measured two attributes: carbon fraction, i.e., relative carbon content, and bulk density, using the laboratory methods described below. Each sample, then, is characterized by two dependent variables (carbon fraction and bulk density) and by four factors: (a) the specific mangrove forest, or location, (b) the sampling site within the mangrove location, (c) the depth at which the sample was extracted from the core, and (d) the coring equipment used to extract that particular sample. In statistical design terms, mangrove location and sampling site nested within location are both random blocks (or random factors), while sediment depth and corer are fixed‐effect factors.

Because the corers did not always penetrate the substrate, not all corers were used at each site. This was especially true for the Russian peat corer, which proved difficult to drive into wet sandy sediments. So, in total, we obtained 52 samples: 16 samples with the soil probe, 16 samples with the open‐faced corer, 14 samples with the Russian peat corer, and six samples with the spade. To compare the bulk densities obtained with the different corers, we did pairwise comparisons of the samples that overlapped between any two coring methods (see Table 1). The soil probe and the open‐faced corer had 15 samples in common, and the Russian peat corer had 13 samples shared with the soil probe and 14 with the open‐faced corer. All corers shared six samples with the spade sampler, as this is the total number of samples that could be extracted with his method. A summary table is presented in Supporting Information with all the samples from La Paz Bay (Appendix S2).

**TABLE 1:** Pairwise comparison among the five different estimation methods for bulk density.

	Soil probe	Open‐faced corer	Russ. Peat corer	Spade sample
Soil probe	—	*r* = .66 (*n* = 15)	*r* = .53 (*n* = 13)	*r* = .92 (*n* = 6)
Open‐faced corer	*b* = 0.90, SE = ±0.11	—	*r* = .60 (*n* = 14)	*r* = .84 (*n* = 6)
Russ. peat corer	*b* = 0.96, SE = ±0.12	*b* = 0.97, SE = ±0.15	—	*r* = .90 (*n* = 6)
Spade sample	*b* = 0.96, SE = ±0.06	*b* = 1.17, SE = ±0.11	*b* = 1.29, SE = ±0.10	—

*Note*: The right upper triangle, above the diagonal, shows the correlation coefficients (*r*) between instruments and number of paired samples (*n*). The lower triangle, below the diagonal, shows the major axis slope from the origin (*b*) and the jackknifed standard error of the slope (SE). All correlations were significant at *p* < .05, with the exception of soil probe versus Russian peat corer that had a significance of *p* = .06. The diagonal cells, shaded in grey, have no values as they correspond to the same corers.

### 2.4 Sample processing and analysis

Upon returning from the field, all samples were placed in a convection oven (Thermo‐Fisher Scientific) to desiccate at 60°C until constant weight was achieved to the nearest tenth of a gram. The dry samples were weighed uncapped in their corresponding glass jar and the dry weight of the sample was obtained by subtracting the weight of the empty jar. The samples were then individually ground using a mortar and pestle. The samples were considered fully homogenized when all particles passed through a 500 μm mesh sieve.

Inorganic carbon in the form of calcium carbonate was removed prior to the sediment analysis through acid fumigation, following Ramnarine et al. (2011): A 300 mg subsample was taken from each fully ground and dried sample and placed into a 20‐ml glass scintillation vial moistened with 150 μl of deionized water. Each uncapped vial was placed in a sealed and vacuumed Pyrex desiccator with a beaker with 100 ml of 12 M hydrochloric acid (HCl). After 72 h of exposure to HCl fumes under vacuum, the beaker containing HCl was removed, and the subsamples flushed with air to clear any residual HCl gas. A small portion of about 9 mg of sampled sediment was placed in a 9 × 6 mm tin capsule, sealed (to prevent leakage), compressed (to remove trapped CO_2_), arranged in a 96‐well microtiter plate, and sent to the University of California Davis (UCD) Stable Isotope Facility, where they were analyzed for carbon fraction using an Elementar‐Vario elemental analyzer (Elementar Analysensysteme GmbH) interfaced to an Isoprime VisION Isotope Ratio Mass Spectrometer (Elementar UK Ltd; see https://stableisotopefacility.ucdavis.edu/carbon‐and‐nitrogen‐solids).

### 2.5 Prediction of bulk density from carbon content

We modeled the inverse functional relationship between sediment bulk density and organic matter content following Stewart et al.'s (1970) mixing model equation:

(1) $$\delta =\frac{\delta_{p}\delta_{m}}{\delta_{m}O+\delta_{p}\left(1-O\right)}$$

where *δ* is the estimated bulk density of the sample, *O* is the proportion of organic matter (or pure peat) in the sediment, *δ*_*p*_ is the bulk density of pure peat, and *δ*_*m*_ is the bulk density of pure mineral sediments. The theory and derivation of this model is provided in Appendix S3. Although the mixing model has been known and used in soil science for over half a century (e.g., Adams, 1973), it has been used for carbon estimates in coastal marshlands and peatlands only in the last decade (Holmquist et al., 2018; Morris et al., 2016). One of the most attractive aspects of this model is that it only has two parameters to be estimated for the fitted function, *δ*_*p*_ and *δ*_*m*_, which correspond to the bulk, self‐packing densities of pure peat and pure mineral sediments, respectively. These parameters have a simple and direct ecological interpretation and can be obtained from regression of bulk density versus carbon fraction data, or from the literature, for the estimation of carbon in mangrove sediments.

If organic matter is measured gravimetrically by loss‐on‐ignition, *O* is the percentage mass that is lost after treatment in the muffle furnace at 450°C. However, if organic carbon is measured with an elemental analyzer, it must be converted into total organic matter. The proportion of carbon‐to‐total organic matter in tropical peat ranges from 40% to 55% (Andriesse, 1988; Craft et al., 1991); it varies according to a multiplicity of factors such as the type of plant material, the content of clay in the sediment, and the hydrology of the lagoon, among others (Atwood et al., 2017). In our own datasets, we found a regression slope between LOI and carbon fraction of 2.2, which implies a 45% proportion of carbon in the sediments' organic matter (see Appendix S4). This value is consistent with those reported in other studies (Atwood et al., 2017; Cinco‐Castro et al., 2022; Ouyang & Lee, 2020; Pribyl, 2010), so we multiplied the proportion of carbon in our samples by a conversion factor *f* = 2.2 to get an estimate of total organic matter. Thus, the model that relates carbon fraction (i.e., relative carbon content *C*_*s*_) to bulk density in peaty mangrove sediments becomes:

(2) $$\delta =\frac{\delta_{p}\delta_{m}}{\delta_{m}\mathrm{fC}+\delta_{p}\left(1-\mathrm{fC}\right)}$$

The carbon density (*D*) in a sediment sample is the product of relative carbon content (*C*) and bulk density (*δ*). As noted by Holmquist et al. (2018), given the relative carbon content in a peaty sediment, and knowing the bulk densities of pure peat (*δ*_*p*_) and pure mineral sediment (*δ*_*m*_), the density of carbon in the sample can be calculated from Equation (2) so that

(3) $$D=\frac{C\delta_{p}\delta_{m}}{\delta_{m}\mathrm{fC}+\delta_{p}\left(1-\mathrm{fC}\right)}\;$$

### 2.6 Estimation of model parameters

Because the mixing model is not linear, to estimate the model parameters (*δ*_*p*_ and *δ*_*m*_), we used nonlinear least‐squares regression with a Gauss–Newton algorithm for parameter search (Nocedal & Wright, 1999), implemented through the *nls* function in the R language (R Core Team, 2022). Because in a nonlinear model the variances are not necessarily additive, a standard ANOVA test is not valid. For this reason, we measured the quality of the fit by means of a lack‐of‐fit test, i.e., a variance ratio test with the variance of the sampling points from the model's predictions in the numerator, and the within‐samples variation, or “pure error” in the denominator (Neill, 1988).

After fitting the mixing model, the residuals of the fitted function were then tested with linear models against other possible predictors of bulk density, such as the random effect of each location, a potential effect of the sites selected within each location, or the depth of the core. The results were summarized in a variance decomposition table, similar to multiple‐regression ANOVAs, with the only difference being that the main explanatory variable, relative carbon content, was fitted by nonlinear estimation, and all other possible factors were tested by linear regression on the residuals.

### 2.7 Model validation with a large dataset

To test whether this model for predicting sediment carbon density from carbon relative content (i.e., carbon fraction) behaves similarly in mangroves from throughout the region, we used a larger dataset of mangrove sediment carbon content and bulk density (Costa et al., 2022). These data are from samples taken at mangrove locations from the Caribbean and Pacific coasts of Panama and throughout the Baja California Peninsula, and from the sediment surface to the maximum depth of corer penetration. The cores were taken with the same Russian peat corer as used in this study, and the samples were processed and analyzed following the same methods, with the exception that the samples from the Caribbean coast of Panama were analyzed by loss on ignition (LOI), with a subset of 20 samples also analyzed using an elemental analyzer to construct a linear calibration curve to relate carbon content to LOI (see Appendix S4).

## 3 RESULTS

### 3.1 Comparison among corers

**FIGURE 3:**
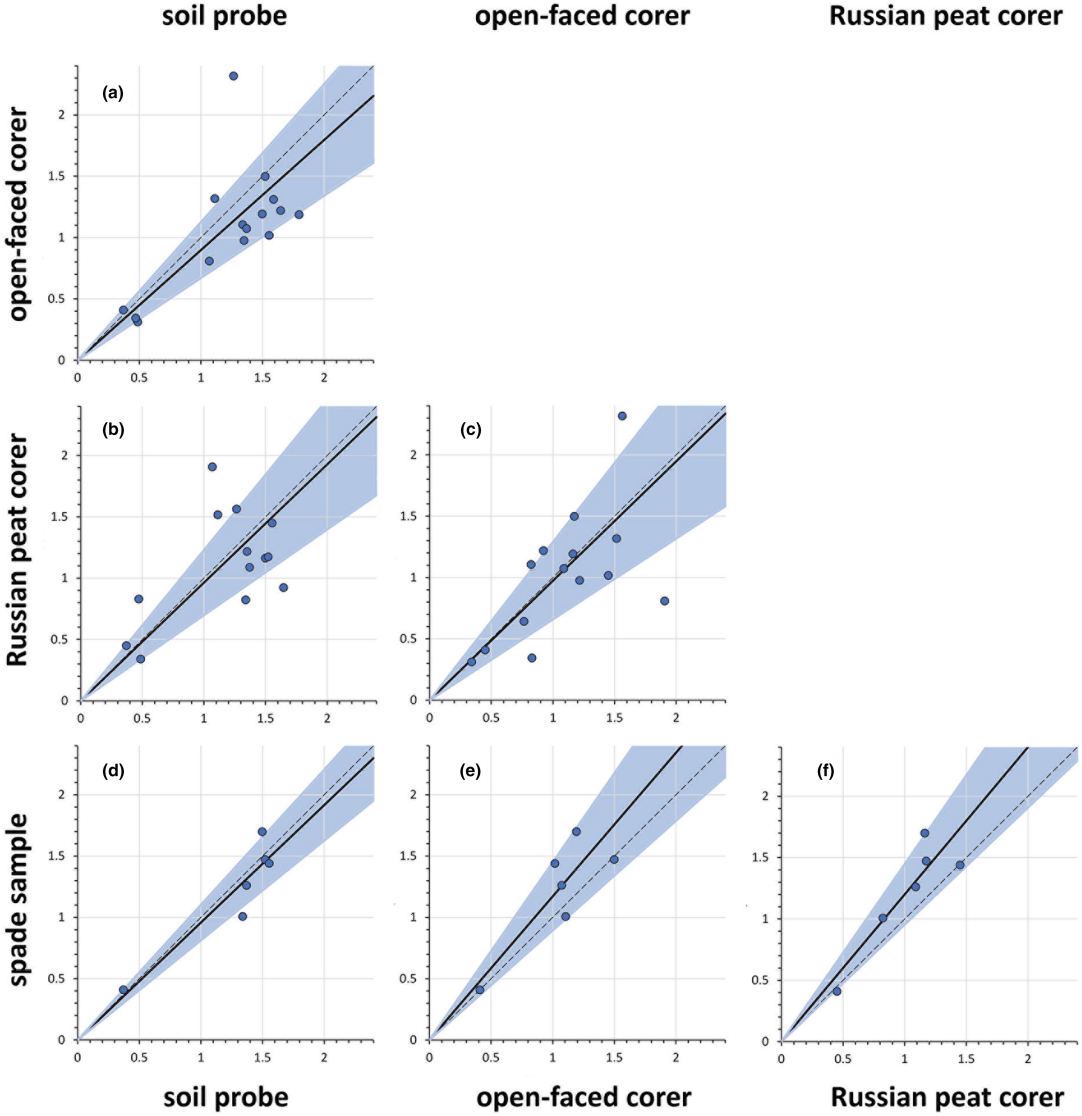
Pairwise major axis regressions between the bulk densities of the three corers plus that from cutout, undisturbed sediment aggregates (spade). The dotted line represents the identity function, and the shaded regions describe the 95% confidence interval. None of the regressions differed significantly in their slope from the identity function (see Table 1 for numeric values of each regression).

The bulk densities estimated by the three corers were significantly correlated with each other and with the bulk density measured from cutout, undisturbed sediment aggregates (Table 1). More importantly, the major axis regression slopes (*b*) between the three corers and the “true” bulk density estimated from the cutout aggregates did not differ significantly from an identity function (i.e., *b* = 1; Figure 3 and Table 1). In short, the bulk densities estimated by the three corers did not differ significantly between corers, or did they differ significantly from the bulk density of undisturbed sediment aggregates.

### 3.2 Relationship between bulk density and carbon content

**FIGURE 4:**
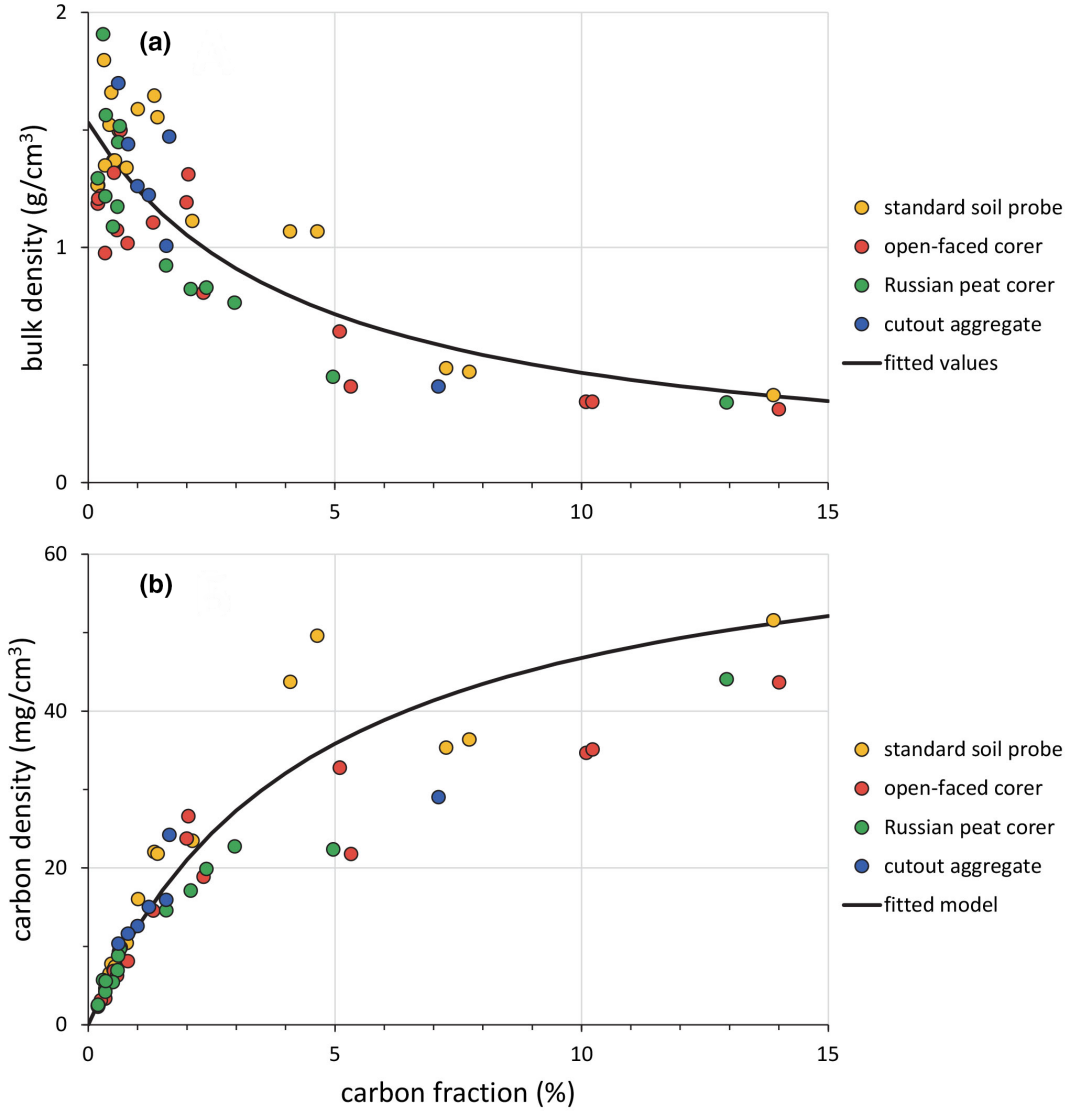
(a) Relationship between bulk density of the La Paz sediment samples and the carbon fraction in the sample. The black curve represents the values fitted by the mixing model. (b) Carbon density (bulk density × carbon fraction) against carbon fraction for the same dataset. The black curve represents the fitted values from the mixing model. Note how data dispersion around the fitted values increases as the organic matter content of the sediment increases.

Using the mixing model, a strong statistical relationship was found between gravimetric bulk density and carbon fraction, i.e., the proportion of carbon in the sediment sample (*r* ^2^ = .715; Figure 4a). The lack‐of‐fit test indicated that the deviation of the mixing model from the mean bulk density at each site was not significantly different from the within‐site variation (*F* = 0.49, *p* = .95). An analysis of variance on the residuals of the nonlinear model found no significant effects of location or sample depth. There was a significant (*p* = .02) effect of site, which can be attributed to the sediment type of each particular site: Mogote site 2, a site with densely clayey sediments, had lower residual bulk densities than the other, nonclayey, sites. There was also a slight but significant (*p* < .01) effect of the choice of corer that had not been detected with the pairwise comparisons: the soil probe and the spade had higher residual bulk densities that the open‐faced or the Russian peat corers. Although significant, these differences are of small quantitative importance in the estimation of bulk density: 71% of the variation in the estimated bulk densities was accounted for by the amount of organic matter in the mixing model, 6% was accounted for by the choice of corer, and 5% by the sedimentology of the site.

Multiplying the fitted bulk densities by the carbon fraction in the sample, an estimate of the carbon density in each sampling site was obtained (Figure 4b). The fit of this model, predicting total carbon as a function of carbon fraction to the same data using gravimetric estimates of bulk density, was good (*r* ^2^ = .865). However, the squared residuals were significantly correlated with predicted carbon content (*r* = .75; *F* = 70.3; df 1, 55; *p* < .0001); a statistical problem that suggests that the dispersion of error in the data using the gravimetric estimate of bulk density increases with the amount of peat in the sediment. Finally, the coefficients of the mixing model were *δ*_*p*_ = 0.135 ± 0.019 and *δ*_*m*_ = 1.530 ± 0.062.

### 3.3 Testing the mixing model on larger datasets

**FIGURE 5:**
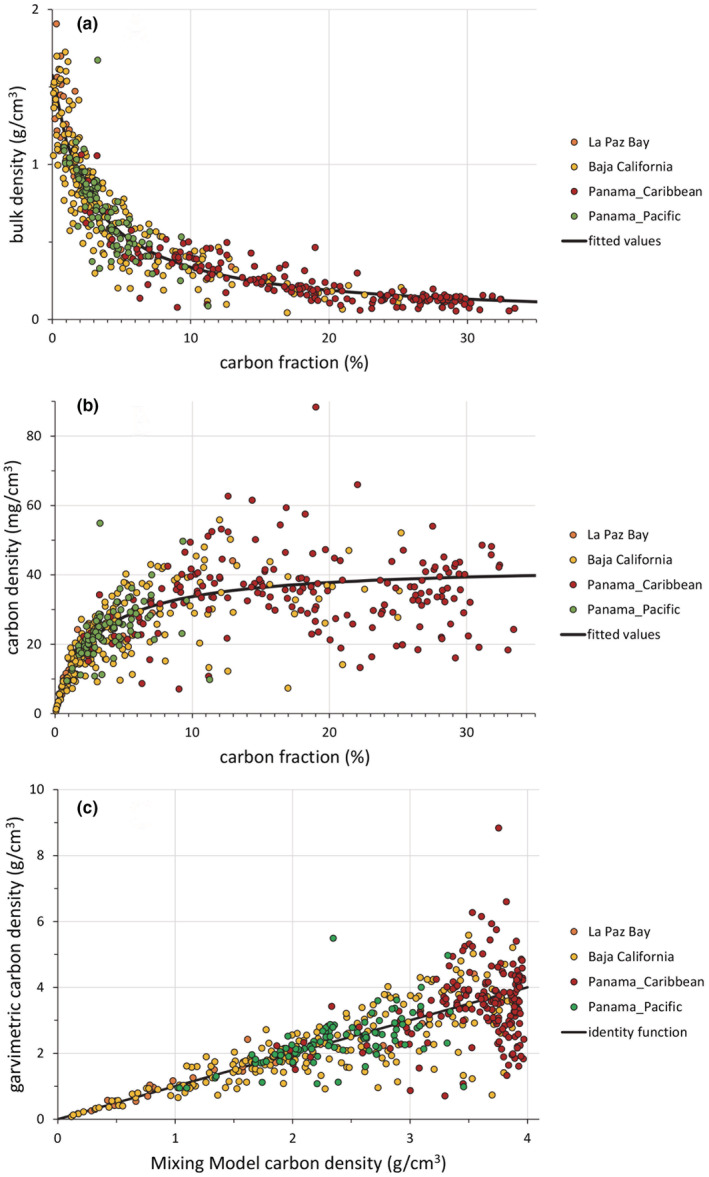
(a) Relationship between bulk density in our pooled dataset (La Paz Bay, Baja California, Panama's Caribbean coast, and Panama's Pacific coast) against carbon fraction in the sample. The black curve represents the values fitted by the mixing model described in Equation (2). (b) Carbon density (bulk density × carbon fraction) against carbon fraction for the same dataset. The black curve represents the fitted values from the mixing model described in Equation (3). (c) Gravimetric carbon fraction versus the mixing model estimation of carbon fraction. Note that, as in Figure 4, data dispersion around the fitted values increases as the sediments increase in their organic matter content.

When the mixing model was tried against the pooled dataset from Costa et al. (2022), a similarly strong relationship was found between the bulk density and the carbon fraction of the sediments. In this case, the carbon fraction predicted 85.5% of the total variation in the bulk density data (*r* ^2^ = .855; Figure 5a). As with the local La Paz dataset, the lack‐of‐fit test indicated that the departure of the mixing model from the within‐carbon‐level means was not significantly different from the pure error (*F* = 0.49, *p* = .95), indicating that the fit is statistically robust.

However, the relationship between carbon fraction and carbon density showed a high dispersion between the predictions of the mixing model and the values calculated using gravimetric estimates of bulk density (*r* ^2^ = .315, Figure 5b), and, as with the previous dataset, the residuals were significantly correlated with the fitted values (*r* = .43, *F* = 112.9; df 1, 494; *p* < .0001). This result suggests, again, that gravimetric bulk density measurements yield statistical estimates of total carbon that are strongly heteroscedastic and dependent on the value of the carbon fraction in the sediment. The coefficients of the mixing model for the pooled dataset were *δ*_*p*_ = 0.082 ± 0.0025 and *δ*_*m*_ = 1.575 ± 0.0322. Note that, because the pooled dataset contained many sites with large amounts of peat, the estimate of *δ*_*p*_ has a much lower standard error and is hence more precise than in the La Paz dataset alone.

## 4 DISCUSSION

### 4.1 Comparison among corers

Despite their differences in diameter and core‐sectioning method, the three corers produced similar and comparable results when compared through pairwise correlations, and slight, quantitatively minor differences when compared using the residuals of the fitted model. When discussing this issue, corer selection in coastal studies such as this one often refers to the tendency for sediment cores to undergo vertical compaction during sampling (Morton & White, 1997). For instance, the open‐faced corer may cause less vertical compaction than the standard soil probe because its larger diameter puts a lower fraction of the sample within proximity to the corer wall. The Russian peat corer is designed to eliminate vertical compaction by closing horizontally around a segment of sediment adjacent to the corer. The fact, however, that no significant or very small differences in bulk density were observed among the corers tested in this study, and the true bulk density estimates obtained from cutting the sediment with a spade suggests that compaction induced by the sampling methods was not substantial in this study of near‐surface wetland sediments. Artificial compaction may increase with depth of coring, so for other applications, such as paleo‐reconstruction, this consideration with regard to corer choice may be more important. Most blue carbon studies focus on the top meter of sediment (Holmquist et al., 2018; Pendleton et al., 2012), so the nondetectable or minor influence of corer choice on bulk density estimates demonstrated in this depth range is of practical applicability to the field.

The main factor in the selection of a corer is possibly the operational difficulties that may be encountered in the field with core penetration and recovery. Because of its smaller diameter size, the soil probe was able to penetrate relatively hard sediments like sand and clay with low amounts of organic matter, while the other two corers often proved difficult to drive into these substrates. In waterlogged, peaty substrates, in contrast, the Russian peat corer worked optimally because it encloses the peat sample in a semi‐cylindrical chamber by cutting laterally once the corer is at the desired depth. The open‐faced corer worked very well in mangrove sediments, but was difficult to operate on the forest edges, sand bars, or the harder upper mudflats. The decision on what corer to use, finally, may depend on the type of substrate and the familiarity of the user with the equipment, but it is important to know that, once a core has been successfully extracted, the type of corer used will not have a large influence on the final estimates.

### 4.2 Relationship between organic carbon content and bulk density

There was a very narrow relationship between the carbon fraction in the sample and its bulk density, which showed a very close fit to the mixing model equation. The model only needs two parameters, the bulk density of pure peat (*δ*_*p*_) and the bulk density of pure mineral sediments (*δ*_*m*_). Other studies (Holmquist et al., 2018; Morris et al., 2016) have fitted the mixing model to coastal wetland data and found values for *δ*_*p*_ and *δ*_*m*_ very close to the ones reported in this study (Table 2), a fact that suggests that the mixing model is a robust and consistent predictor of bulk density in waterlogged sediments. Excluding the parameters from La Paz Bay (which were included in the larger, pooled dataset), the mean values for *δ*_*p*_ and *δ*_*m*_ in this study and two other published ones were 0.09 ± 0.009 and 1.75 ± 0.217, respectively. Although more studies are necessary to confirm these results, it seems clear that using the mixing model's equation with parameter values *δ*_*p*_ = 0.085 and *δ*_*m*_ = 1.65, the carbon fraction, or organic matter fraction, will yield a good, conservative estimate of the sample's bulk density.

**TABLE 2:** Values of the bulk density (BD) parameters of the mixing model reported in this study (pooled dataset), in Holmquist et al. (2018) and Morris et al. (2016).

	Pooled dataset		Holmquist et al.		Morris et al.	
	Value	SE	Value	SE	Value	SE
BD of peat	0.081	±0.002	0.098	±0.001	0.085	±0.001
BD mineral sediment	1.575	±0.032	1.670	±0.025	1.990	±0.028

### 4.3 Gravimetry or carbon fraction? Choosing the best estimate of bulk density

The previous analysis shows that bulk density can be reliably estimated from carbon fraction data if adequate parameters are used. The question that follows is how precision and accuracy vary between the two approaches. In order to test this—and taking advantage of the fact that no significant differences were found in the bulk densities estimated by each of the three corers—we took each corer within each sampling site as a replicate of the site's bulk density estimation, and we ran a linear model taking gravimetric bulk densities as the dependent variable, the site as the predictor, and the three corers as replicates within each site. We then performed the same analysis, taking carbon‐based estimates of bulk density as the dependent variable. Because in a linear model with this design the residual term in the ANOVA is a measure of within‐site variation, we checked which of the estimates of bulk density gave a proportionally lower residual term, as a measure of replicability and consistency in the results. We found that the within‐sites variation for the gravimetric estimate was 24% of the total observed sum of squares, while the within‐sites variation for the carbon‐based estimate was only 15% of the total variation, proportionally much less. The differences between the two within‐site variation terms were significant according to a variance ratio test (*F* = 2.32, df 33, 33; *p* = .009). We can conclude, then, that the carbon‐based estimate of bulk density has a lower variation between replicate measures.

### 4.4 The challenge of heteroscedasticity in total carbon estimation

Although the functional relationship between carbon fraction and gravimetric bulk density is strong, the product of the two variables to calculate carbon density in the sediment shows a wide, funnel‐shaped dispersion of the data points that increase as the sediments become richer in organic matter. We argue here that this phenomenon is a result of the way errors propagate in a product. In its simplest form, if a variable *z* is the product of two variables *x* and *y* so that *z* = *xy*, then it follows that d*z*/d*x* = *y*. Approximating the differential d*x* with its small increment equivalent Δ*x*, we can write Δ*z* = yΔ*x*. That is, a small error (*ε*_*x*_ = Δ*x*) in one of the variables intervening in the product will be amplified by the value of the other variable in the product so that *ε*_*z(x)*_ = *yε*_*x*_ and *ε*_*z(y)*_ = *xε*_*y*_. This implies that in a model based on the product of two variables with independent random errors, the dispersion in the model will increase with the values of the intervening variables. This simple conclusion is in agreement with statistical theory: It is a well‐known fact in statistics (e.g., Bohrnstedt & Goldberger, 1969; Goodman, 1960) that the variance of the product of two independent variables *x* and *y* with random, independent errors is *V*(*xy*) = *E*(*y*)^2^ *V*(*x*) + *E*(*x*)^2^ *V*(*y*) + *V*(*x*)*V*(*y*). Note that the variance of each variable (*V*) propagates onto the calculated product multiplied by the square of the expected value (*E*) of the other variable, a fact that predicts, again, that the dispersion in the model will increase as the value of the intervening variables increases (Appendix S5a). If the variables in the product are not independent but correlated, the formula becomes more complex because additional terms must be added to correct for the effect of correlation on the product (Goodman, 1960), but the first two terms (*E*(*y*)^2^ *V*(*x*) and *E*(*x*)^2^ *V*(*y*)) are still the main contributors to the total variance. In short, when two variables with independent, random errors are multiplied, the dispersion in the data will increase as the main predictor variable increases, which is what is observed in the calculation of total carbon using the product of gravimetric bulk density and carbon fraction.

If, on the other hand, bulk density is estimated directly from the value of carbon fraction using the mixing model equation *D* = *f*(*C*), where *f*(*C*) is the mixing model equation described in Methods (Equation 3), then a differential equation model can be used to estimate how an error in the estimation of carbon fraction will propagate onto the estimation of total carbon density. By definition, d*D*/d*C* = *f*'(*C*). Approximating the differential d*C* with its small increment equivalent Δ*C*, we can write Δ*D* ≅ *f*'(*C*)Δ*C*. That is, a small error in the estimation of carbon fraction (*ε*_*C*_ = Δ*C*) will propagate onto the estimation of total carbon density multiplied by the first derivative, or slope, of the *D* versus *C* function. Because the slope of the function decreases for high values of *C*, then it follows that for sites with high carbon fraction (i.e., peaty sediments), the error in the estimation of total carbon density will be lowest. Furthermore, because the first derivative of the model (Equation 3) can be shown to be $\delta_{C}^{2}/\delta_{p}$, where *δ*_*C*_ is the bulk density predicted by the mixing model for an estimated carbon fraction *C*, and *δ*_*p*_ is the bulk density of pure peat, it is easy to see that as the carbon fraction in the sediment increases, *δ*_*C*_ will decrease according to the model, and the dispersion in the estimated values will decrease (Appendix S5b).

### 4.5 Accuracy vs. precision in carbon density estimation

**FIGURE 6:**
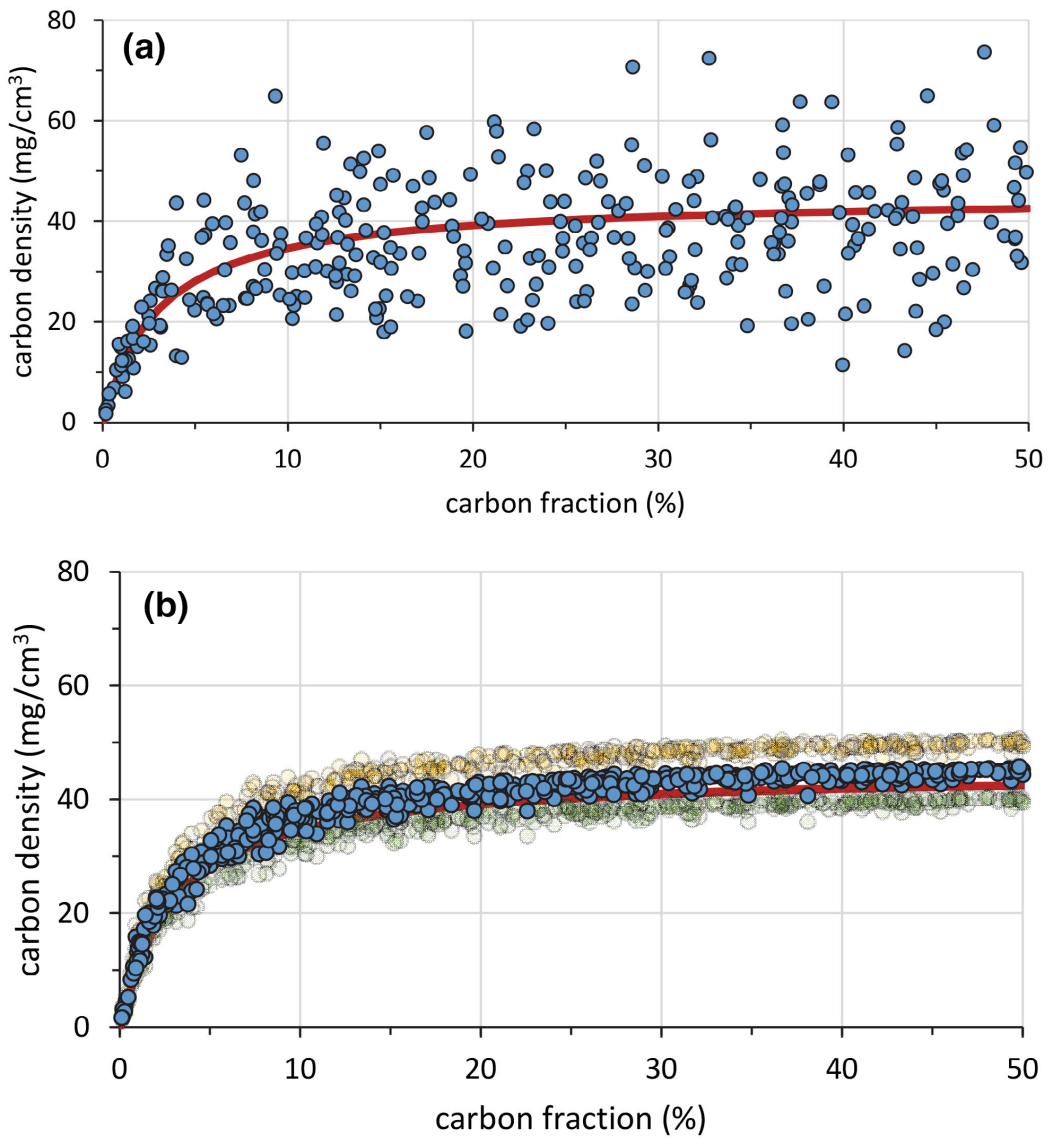
Monte Carlo simulation for sample values of carbon fraction and bulk density with normalized, independent random errors. (a) Carbon density estimated by multiplying the carbon fraction by the bulk density of the randomized variables (Equation 2). (b) Carbon density estimated using the mixing model with carbon fraction as the sole input (Equation 3). This last simulation was done with parameter values for the mixing model of *δ*_*m*_ = 1.75 g cm^−3^ and *δ*_*p*_ = 0.09 g cm^−3^ (the mean of published values for the parameters; blue dots). In order to assess the sensitivity of the carbon density estimation to the parameters, we repeated the simulation with *δ*_*m*_ = 2.00 g cm^−3^ and *δ*_*p*_ = 0.10 g cm^−3^ (the upper limit of reported values, pale yellow dots), and with *δ*_*m*_ = 1.50 g cm^−3^ and *δ*_*p*_ = 0.08 g cm^−3^ (the lower limit of reported values, pale green). In both graphs, the true data values are represented by the continuous red line. See Appendix S5 for more information.

Adding to the preceding algebraic derivation, the heteroscedasticity of the data points when using the gravimetric estimate of bulk density was also tested empirically using a Monte Carlo simulation as described in detail in Appendix S5c. By introducing normalized random errors to simulated sample values for carbon fraction and bulk density, we estimated carbon density by (a) multiplying the carbon fraction by the bulk density of the randomized samples (Equation 2; Figure 6a), and (b) using the mixing model with carbon fraction as the sole input (Equation 3; Figure 6b). As with the real data, the dispersion in the estimation of carbon density when using carbon fraction and the gravimetric estimate of bulk density increased as the sediment became richer in organic matter while the relative error when using carbon fraction only to estimate bulk density through the mixing model decreased as the sediment became more peaty.

It seems clear from the above reasoning that the precision of the carbon density model solely based on carbon fraction is much higher than the estimate using the product of carbon fraction and gravimetric bulk density. Indeed, the dispersal of data points when predicting total carbon density from gravimetric bulk density data is so large that Holmquist et al. (2018) decide to base their carbon‐density mapping at a continental level using the binary categories of organic‐ and mineral‐dominated sediments. The product of gravimetric bulk density and carbon fraction has an extremely high data dispersion and hence is very imprecise, but it is important to note that the product estimator is unbiased, in the sense that the expected value of a sample is the true value in the field, and, in the strict sense of the statistical definition, it is accurate. In contrast, the estimation based solely on carbon fraction has a very high precision, but the final estimate of total carbon density can be biased because it depends on the accuracy with which the two parameters of the model, *δ*_*p*_ and *δ*_*m*_, have been estimated (see Figure 6b). Thus, the accuracy of the estimate of total carbon based on the mixing model depends very strongly on the accuracy with which *δ*_*p*_, the bulk density of pure peat, and *δ*_*m*_, the bulk density of pure mineral sediment, are estimated.

## 5 CONCLUSIONS

Research in the last two decades has revealed the large role played by mangroves, seagrass beds, and marshlands in CO_2_ sequestration and carbon immobilization in their sediments (Chmura et al., 2003; Lovelock & Duarte, 2019; Rockström et al., 2021). The choice of corer to sample mangrove sediments does not seem to have much influence on the final estimates of carbon density. The main factor in the selection of a corer is more related to the operational difficulties that each corer may have in different types of sediments than to the accuracy of the estimate.

The bulk density of a core sample can be estimated gravimetrically, by cutting and dry weighing a segment of the core, but it can also be estimated from the carbon fraction in the sample, using the mixing model equation. Because of the multiplication of errors in a product of two variables subject to random sampling error, when using gravimetric estimates of bulk density, the dispersion of the data points in the estimation of total carbon density rises rapidly as the amount of carbon in the sediment increases. For this reason, the estimation of carbon densities in peaty sediments using gravimetric bulk densities can be very imprecise. Historically, the gravimetric approach has dominated, followed by loss‐on‐ignition analysis, possibly because it is less costly than analyzing all samples on an elemental analyzer. Our study demonstrates, however, that even with loss‐on‐ignition, the mixing model can be used with increased precision.

The estimation of total carbon density using only the carbon fraction as a predictor is very precise, especially in sediments rich in organic matter. This method, however, depends critically on the accurate estimation of the two parameters of the mixing model (the bulk density of pure peat and the bulk density of pure mineral sediment) and on the conversion factor from organic carbon fraction to organic matter. If these parameters are not estimated with accuracy, the calculation of total carbon density can be biased. It is recommendable to use relatively low values of *δ*_*p*_ and *δ*_*m*_, and a relatively high conversion factor of carbon fraction‐to‐LOI (implying that the proportion of carbon in organic matter is less than 50%), so that the estimates of carbon density are conservative.

In practical terms, these findings open the door to simpler and more precise estimations of blue carbon in mangrove sediments. They also open the door to the possibility of using pre‐existing data containing elemental carbon or organic matter assessments in coastal lagoon sediments for the precise estimation of blue carbon storage, even if data on bulk density are lacking. We believe that the use of the mixing model in carbon storage estimations can detonate many new assessments of blue carbon storage with a simpler, quicker, and statistically more robust method.

## AUTHOR CONTRIBUTIONS

**Paula Ezcurra:** Conceptualization (equal); data curation (equal); formal analysis (equal); methodology (equal); project administration (equal); writing – original draft (equal); writing – review and editing (equal). **Matthew T. Costa:** Conceptualization (equal); data curation (equal); formal analysis (equal); methodology (equal); writing – original draft (equal); writing – review and editing (equal). **Octavio Aburto‐Oropeza:** Conceptualization (equal); data curation (equal); formal analysis (equal); funding acquisition (equal); methodology (equal); writing – original draft (equal); writing – review and editing (equal). **Paula Sternberg‐Rodríguez:** Conceptualization (equal); data curation (equal); formal analysis (equal); methodology (equal); project administration (equal); writing – original draft (equal); writing – review and editing (equal). **Exequiel Ezcurra:** Conceptualization (equal); data curation (equal); formal analysis (equal); funding acquisition (equal); methodology (equal); writing – original draft (equal); writing – review and editing (equal).

## FUNDING INFORMATION

We thank the Aburto lab at SIO and the Climate Science Alliance for providing funding for this research project.

## CONFLICT OF INTEREST

The authors declare no conflict of interest.

## Supporting information


Appendix S1–S5.

## Data Availability

The datasets used for this study are available at the following link: https://doi.org/10.6086/D1TX0T.
