# Supplementary material for: Precision of mangrove sediment blue carbon estimates and the role of coring and data analysis methods
Source: Ecol Evol. 2022 Dec 25;12(12):e9655. doi: 10.1002/ece3.9655 (PMC9790802; doi:10.1002/ece3.9655)
Supplement: Supplementary file 1 — Appendix S1–S5. [file ECE3-12-e9655-s001.docx]

Paula Sternberg-Rodríguez, Paula Ezcurra, Matthew T. Costa, Octavio Aburto-Oropeza, and Exequiel Ezcurra. “Precision of mangrove sediment blue carbon estimates and the role of coring and data analysis methods.” *Ecology and Evolution*

**Appendix S1. Russian peat corer cross-sectional area**

The Russian peat corer’s inner diameter is 2.5 cm, and it samples sediment in a half-circular cross-section, with the first 2 mm of that half-circle, starting from the origin, blocked from including sample by the area taken up by the 4 mm-wide steel plate around which the sampler turns. The cross-sectional area of the entire half-circle is *π*(2.5 cm)^2^/2 = 9.81 cm^2^, and the area of the half of the plate intruding into the sampling chamber is equal to half the width of the plate (0.2 cm) times the inner diameter of the corer (5 cm), or 0.2 cm × 5 cm = 1 cm^2^. Thus, the total core cross-sectional area equals 9.81 cm^2^ – 1 cm^2^ = 8.81 cm^2^.

**Appendix S2. Sampling design and sample values for La Paz Bay dataset**

| spade | %C | 0.609 |  | 1.581 | – | – | – | 1.645 | – | – | 1.228 | – | – | 7.099 | – | 0.807 | – |  |
| --- | --- | --- | --- | --- | --- | --- | --- | --- | --- | --- | --- | --- | --- | --- | --- | --- | --- | --- |
|  | b. dens. | 1.699 |  | 1.007 | – | – | – | 1.472 | – | – | 1.223 | – | – | 0.409 | – | 1.44 | – |  |
| Russian peat corer | %C | 0.195 |  | 2.078 | 0.637 | 0.299 | 2.395 | 0.593 | – | – | 0.5 | 0.345 | 1.579 | 4.964 | 12.942 | 0.608 | 0.355 | 2.967 |
|  | b. dens. | 1.295 |  | 0.823 | 1.516 | 1.907 | 0.83 | 1.174 | – | – | 1.089 | 1.217 | 0.923 | 0.45 | 0.34 | 1.449 | 1.563 | 0.766 |
| open-faced corer | %C | 1.992 | 0.194 | 1.316 | 0.521 | 2.336 | 10.092 | 0.658 | 2.028 | – | 0.584 | 0.343 | 0.256 | 5.323 | 14.004 | 0.798 | 0.203 | 5.098 |
|  | b. dens. | 1.192 | 1.187 | 1.106 | 1.318 | 0.808 | 0.343 | 1.499 | 1.311 | – | 1.074 | 0.976 | 1.219 | 0.409 | 0.312 | 1.018 | 1.207 | 0.643 |
| soil probe | %C | 0.618 | 0.318 | 0.777 | 2.11 | 4.092 | 7.724 | 0.431 | 1.009 | 0.468 | 0.51 | 0.344 | 1.338 | 13.89 | 7.255 | 1.403 | 0.199 | – |
|  | b. dens. | 1.497 | 1.796 | 1.339 | 1.112 | 1.068 | 0.471 | 1.522 | 1.589 | 1.659 | 1.37 | 1.349 | 1.646 | 0.371 | 0.487 | 1.554 | 1.265 | – |
| depth |  | upper | middle | upper | middle | lower | upper | upper | middle | lower | upper | middle | lower | upper | middle | upper | middle | lower |
| site |  | 1 | 1 | 2 | 2 | 2 | 3 | 1 | 1 | 1 | 2 | 2 | 2 | 1 | 1 | 2 | 2 | 2 |
| location |  | Conchalito | Conchalito | Conchalito | Conchalito | Conchalito | Conchalito | Mogote | Mogote | Mogote | Mogote | Mogote | Mogote | Enfermeria | Enfermeria | Enfermeria | Enfermeria | Enfermeria |
| ID |  | C1S1 | C1S2 | C2S1 | C2S2 | C2S3 | C3S1 | M1S1 | M1S2 | M1S3 | M2S1 | M2S2 | M2S3 | X1S1 | X1S2 | X2S1 | X2S2 | X2S3 |

**Appendix S3. Prediction of bulk density from carbon content: the Mixing Model**

Half a century ago, Stewart et al. (1970) described an inverse functional relationship between soil apparent density and organic matter content. They modeled this relationship assuming that the bulk densities of pure organic matter and pure mineral matter are constant and that, in a mixture, the volumes occupied by the organic and mineral components are additive. If we define the proportional mass of organic matter in a mangrove sediment sample as *O* (a value that varies between 0 and 1), then the relative volume occupied by organic matter in a sample is *O*/*δ_p_* , where *δ_p_* is the bulk density of pure organic matter in g.cm^-3^. It follows that the proportional mass of mineral particles in the sample will be equal to (1 – *O*), and hence the relative volume occupied by mineral particles in the sample is (1 – *O*)/*δ_m_* , where *δ_m_* is the bulk density of pure mineral sediments. The sum of both components (*O*/*δ_p_* + (1 – *O*)/*δ_m_*), expressed in cm^3^/g, will give an estimate of the volume occupied jointly by the two fractions in a mixture. The inverse of this calculated joint volume will yield an overall estimation of the apparent density of the sediment sample containing a proportion *O* of peat, so that

$\delta=\frac{1}{\left( \frac{O}{\delta_{p}} \right)+\left( \frac{1-O}{\delta_{m}} \right)}$ (1)

where *δ* is the estimated bulk density of the sample. Because this model assumes that soil bulk volume equals the summed bulk volumes of organic and mineral components, it also assumes that peat accumulation in the sediment creates a volume expansion of the mineral matter equal to its own bulk volume (Adams, 1973). Although this hypothesis may not hold strictly in agricultural soils, where soil structure may vary as a result of farming activities like tilling or trampling by grazing animals, the model has been used with success in marshlands and peatlands under the name “Ideal Mixing Model” (Morris et al. 2016, Holmquist et al. 2018). One of the most attractive aspects of this model is that it only has two parameters to be estimated for the fitted function, *δ_p_* and *δ_m_*, which correspond to the bulk, self-packing densities of pure peat and pure mineral sediments, respectively. These parameters have a simple and direct ecological interpretation and can be obtained from the literature for the estimation of carbon in mangrove sediments without having to estimate them through regression on large datasets.

The independent variable *O* is the proportion of peat in the sediment. If organic matter is measured gravimetrically by loss-on-ignition, *O* is simply the percentage mass that is lost after high-temperature treatment in the muffle furnace. However, if organic carbon is measured with an elemental analyzer, it must be converted to total organic matter to incorporate it into the Mixing Model. As explained in Appendix S3, we found a conversion factor (*f*) of carbon fraction to organic matter of 2.2. Thus, the model that relates carbon fraction (i.e., relative carbon content *C_s_*) to bulk density in peaty mangrove sediments becomes:

$\delta=\frac{1}{\left( \frac{fC}{\delta_{p}} \right)+\left( \frac{1-fC}{\delta_{m}} \right)}$ (2)

where *f* = 2.2. This equation can also be written as:

$\delta=\frac{\delta_{p}\delta_{m}}{\delta_{m}fC+\delta_{p}(1-fC)}$ (3)

The carbon density (*CD*) in a sediment sample *s* is the product between the relative carbon content in the sample (*C*) and the bulk density of that sample (*δ*). As noted by Holmquist et al. (2018), given the relative carbon content in a peaty sediment, and knowing the bulk densities of pure peat (*δ_p_*) and of pure mineral sediment (*δ_m_*), the mass of carbon in the sample, i.e., the carbon density, can be calculated from Eq. 2 so that

$CD=\frac{C}{\left( \frac{fC}{\delta_{p}} \right)+\left( \frac{1-fC}{\delta_{m}} \right)}$ (4)

or, alternatively, from Eq. 3 so that

$CD=\frac{C\delta_{p}\delta_{m}}{\delta_{m}fC+\delta_{p}(1-fC)}$ (5)

**Appendix S4. Converting carbon fraction to organic matter**

The relationship between total organic matter (estimated as loss-on-ignition, or LOI), and percentage organic carbon in the sample (C, or carbon fraction) ranges from 40 to 55% according to different estimations (Andriesse 1988, Craft, Seneca, and Broome 1991); that is, the C-to-LOI conversion factor ranges between 1.8 and 2.5. We tested the relationship between LOI and C using Costa’s (2019) database described in the Methods section of this paper. We used 20 mangrove sediment samples from Panama’s Caribbean coast where coupled measurements of both C and LOI had been made and estimated the conversion factor from the slope of the LOI-to-C linear regression line (Fig. S1).

| 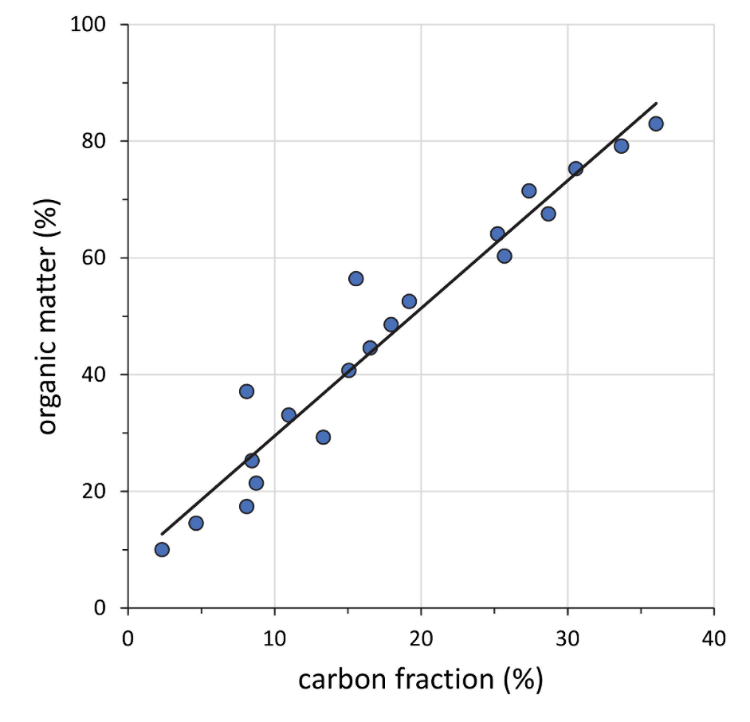 |
| --- |
| Figure S1. Linear relationship predicting organic matter content (loss-on-ignition) in mangrove sediments from the carbon fraction estimated from elemental analysis (*r* = 0.97, df 18, *P* < 0.0001; slope *b* = 2.18 ±0.13). |

LOI and C were highly correlated (*r* = 0.97, df 18, *P* < 0.0001), with a slope of 2.18 ±0.13. This value is very close to other C-to-LOI conversion factors reported in the literature. For example, based on an extensive review of literature on soil science, Pribyl (2010) proposed a conversion factor of 2.0 for soil organic matter. Kauffman and Donato (2012) and Atwood et al. (2017) used a factor of 2.07 to estimate total organic matter from carbon fraction in mangroves. Ouyang and Lee (2020) compiled a database of 1534 observations on coupled measurements of C and LOI in mangrove sediments from 52 countries and found a LOI:C relationship of 2.7 in peaty soils. Finally, Cinco-Castro et al. (2022) reported C and LOI values for four mangrove zones in the Celestún Lagoon, Yucatan. Their ratios ranged from 1.9 to 2.3, with a mean value of 2.1. Thus, based on our own results and their congruence with those reported in the literature, we chose a C-to-LOI conversion factor of 2.2.

**References to Appendix S4**

Atwood, T. B., R. M. Connolly, H. Almahasheer, P. E. Carnell, C. M. Duarte, C. J. E. Lewis, X. Irigoien, J. J. Kelleway, P. S. Lavery, P. I. Macreadie, O. Serrano, C. J. Sanders, I. Santos, A. D. L. Steven, and C. E. Lovelock. 2017. “Global patterns in mangrove soil carbon stocks and losses.” *Nature Climate Change* **7**: 523–528. doi:10.1038/NCLIMATE3326

Cinco-Castro, S., J. Herrera-Silveira, and F. Comín. 2022. “Sedimentation as a support ecosystem service in different ecological types of mangroves.” *Frontiers in Forests and Global Change* **5**:733820. doi:10.3389/ffgc.2022.733820

Kauffman, J.B., V.B. Arifanti, I. Basuki, S. Kurnianto, N. Novita, D. Murdiyarso, D.C. Donato, and M.W. Warren. 2016. *Protocols for the Measurement, Monitoring, and Reporting of Structure, Biomass, Carbon Stocks and Greenhouse Gas Emissions in Tropical Peat Swamp Forests*. Center for International Forestry Research (CIFOR), Bogor, Indonesia. doi:10.17528/cifor/006429

Ouyang, X., and S. Y. Lee. 2020. “Improved estimates on global carbon stock and carbon pools in tidal wetlands.” *Nature Communications* **11**: 317. doi:10.1038/s41467-019-14120-2

Pribyl, D. W. 2010. “A critical review of the conventional SOC to SOM conversion factor.” *Geoderma* **156**: 75–83. doi:10.1016/j.geoderma.2010.02.003

**Appendix S5. Data dispersion and prediction error**

1. **Distribution of errors when using the gravimetric estimate of bulk density**

We will define the bulk density of a sediment as *δ*, and the carbon fraction, or relative carbon content, as *C*. The carbon fraction in a sample will be $\hat{C}=C\pm\varepsilon$, where *ε* is the sampling error in estimating carbon fraction. Similarly, the sample bulk density can be defined as $\hat{\delta}=\delta\pm\varphi$, where *ϕ* is the sampling error in bulk density. Following the traditional approach for the estimation of total carbon density (*D*), we will define $\hat{D}=\hat{C}. \hat{\delta}$, which can be rewritten as $\hat{D}=\left( C\pm\varepsilon\right)\left( \delta\pm\varphi\right).$ Opening the product of the two binomials, we get $\hat{D}=C\delta\pm C\varphi\pm\delta\varepsilon\pm\varepsilon\varphi$. By definition *D* = *Cδ*, so we can rewrite the previous equation as $\hat{D}=D\pm\mathrm{err}(CD)$, where err(*D*) = ±*Cϕ* ±*δε* ±*εϕ*. The addition of all possible combinations of ± signs in err(*D*) is equal to zero, so *E*(err(*D*)) = 0 and hence $E\left( \hat{D} \right)=D$, indicating that $\hat{D}$ is an unbiased estimator of carbon density.

The variance of total carbon density is equal to the expected value of the squared residuals: *V*(*D*) = *E*[err(*D*)^2^]. Solving the equation for err(*D*) squared, we get *V*(*D*) = *C*^2^*ϕ*^2^+*δ*^2^*ε*^2^+*ε*^2^*ϕ*^2^+2*Cεϕ*^2^+2*δε*^2^*ϕ*+2*Cδεϕ* (see Bohrnstedt and Goldberger 1969 for a detailed derivation). Neglecting the terms of lesser numerical value, an approximate formula for the variance of the *C*× *δ* product can be written as *V*(*D*) ≅ *C*^2^*ϕ*^2^ + *δ*^2^*ε*^2^ + 2*Cδεϕ* (Goodman 1960). Furthermore, although carbon fraction and bulk density are negatively correlated, the functional relationship is convex and tends to horizontalize at high values of carbon fraction. That is, in highly organic soils bulk density tends to become constant as it approaches *δ_p_*, and the correlation between *C* and *δ* decreases. Under those conditions, the term *Cδεϕ* tends to zero and the variance estimator is driven largely by the two terms *C*^2^*ϕ*^2^ and *δ*^2^*ε*^2^ so that *V*(*D*) ≅ *C*^2^*ϕ*^2^ + *δ*^2^*ε*^2^. Now, if the error in the estimation of *C* and *δ* is a fixed relative error, proportional to the value of the variable, then we can write *ε* = *Cp* and *ϕ* = *δq*, where *p* is the relative error in the estimation of carbon fraction and *q* is the relative error in the estimation of gravimetric bulk density. Then, the (approximate) equation for the variance in the estimation of carbon density becomes *V*(*D*) ≅ *C*^2^*δ*^2^(*p* + *q*)^2^. Recalling that *D* = *Cδ*, we can write the final equation for the variance of carbon density as *V*(*D*) ≅ *D*^2^(*p* + *q*)^2^. It follows then that the standard error, or mean deviate, of the carbon density estimate can be written as:

*SE*(*D*) ≅ *D* (*p* + *q*) (1)

This reasoning shows that, when estimating carbon density as the product of carbon fraction and gravimetric bulk density, the dispersion of the data points will increase in direct proportion to the amount of organic matter in the sediment and the method will become less precise as the estimation moves from mineral to peaty sediments.

The fact that the variance of the product between two variables is expected to increase as the value of the intervening variables increases is well known in statistical theory (Goodman 1960; Bohrnstedt and Goldberger 1969). A product of two variables may show heteroscedasticity even if the intervening variables are homoscedastic. For the purpose of this study, what is important in these derivations is the fact that, even if the original errors in the estimations of bulk density (*δ*), and carbon fraction (*C*) are uniformly distributed, the statistical theory predicts that the error in the product of both variables will increase as the value of the predictor variables increases. In simple terms, this means that the estimation of total carbon density in a sediment will show a large dispersion of the data points in carbon-rich sediments, as is indeed the case in our field observations.

**(b) Distribution of errors when using carbon-based estimate of bulk density**

To estimate total carbon density (*D*) using the Mixing Model estimate of bulk density we apply Eq. 3 described in the methods section:

$$D=\frac{C\delta_{p}\delta_{m}}{\delta_{m}fC+\delta_{p}(1-fC)}.$$

Applying the quotient rule and the chain rule, the first derivative of this equation is:

$$\frac{dD}{dC}= \frac{\delta_{p}\delta_{m}}{\delta_{m}fC+\delta_{p}(1-fC)}-\frac{{C\delta}_{p}\delta_{m}}{{{(\delta}_{m}fC+\delta_{p}(1-fC))}^{2}}{(f\delta}_{m}-{f\delta}_{p}).$$

Now we extract a common factor, to obtain:

$$\frac{dD}{dC}= \frac{\delta_{p}\delta_{m}}{\delta_{m}fC+\delta_{p}(1-2C)}\left[ 1-\frac{{fC(\delta}_{m}-\delta_{p})}{\delta_{m}fC+\delta_{p}(1-fC)} \right].$$

And we find a common denominator for the sum in parentheses:

$$\frac{dD}{dC}= \frac{\delta_{p}\delta_{m}}{\delta_{m}fC+\delta_{p}(1-fC)}\left[ \frac{{\delta_{m}fC+\delta_{p}\left( 1-fC \right)-fC(\delta}_{m}-\delta_{p)}}{\delta_{m}fC+\delta_{p}(1-fC)} \right].$$

We open all parentheses in the numerator:

$$\frac{dD}{dC}= \frac{\delta_{p}\delta_{m}}{\delta_{m}fC+\delta_{p}(1-fC)}\left[ \frac{{{fC\delta}_{m}+\delta_{p}-fC\delta_{p}-fC\delta}_{m}+{fC\delta}_{p}}{\delta_{m}fC+\delta_{p}(1-fC)} \right].$$

And eliminate all elements that cancel out:

$$\frac{dD}{dC}= \frac{\delta_{p}\delta_{m}}{\delta_{m}fC+\delta_{p}(1-fC)}\left[ \frac{\delta_{p}}{\delta_{m}fC+\delta_{p}(1-fC)} \right].$$

Now multiplying both the numerator and denominator by *δ_m_* we obtain:

$$\frac{dD}{dC}= \frac{1}{\delta_{m}}\left( \frac{\delta_{m}\delta_{p}}{\delta_{m}fC+\delta_{p}(1-fC)} \right)^{2}.$$

Finally, recalling that, according to the mixing model, $\delta=\frac{\delta_{m}\delta_{p}}{\delta_{m}fC+\delta_{p}(1-fC)}$ , we can write the first derivative in simplified form as:

$$\frac{dD}{dC}= \frac{\delta^{2}}{\delta_{m}} .$$

Approximating the differential d*C* with its small increment equivalent Δ*C*, we can write the equation as:

$$\Delta\left( D \right)\cong\frac{\delta^{2}}{\delta_{m}}\Delta C ,$$

or, using the notation employed in the previous section:

$$\mathrm{err}\left( D \right)\cong\frac{\delta^{2}}{\delta_{m}}\varepsilon.$$

As in the previous section, if the error in the estimation of *C* is a fixed relative error, proportional to the value of the variable, then we can write *ε* = *Cp*, where *p* is the relative error in the estimation of carbon fraction. Then, the equation for the variance in the estimation of carbon density becomes *V*(*D*) ≅ *C*^2^(*δ*^4^/*δ_m_^2^*)*p*^2^. Recalling that *D* = *Cδ*, we can write the final equation for the variance of carbon density as *V*(*D*) ≅ *D*^2^(δ^2^/δ_m_^2^)*p*^2^. The standard error, or mean deviation, of the carbon density estimate is then:

$\mathrm{SE}\left( D \right)\cong D\frac{\delta}{\delta_{m}}p$ (2)

Note that the coefficient *δ*/*δ_m_* is always less that one and —because *δ* and C are negatively correlated— it decreases as *C* increases. So, in contrast with the previous section, the above equation shows that, if carbon density is estimated directly from the carbon fraction using the mixing model, the relative dispersion of the data points around the predicted function will decrease in direct relation to the amount of organic matter in the sediment and the method will become more precise as the estimation moves from mineral to peaty sediments.

**(c) Testing the theory of errors with a Montecarlo simulation**

In the previous two sections we have shown that (a) the mean relative error of the estimation of carbon density in a sediment when using carbon fraction and a gravimetric estimate of bulk density will increase as the sediment becomes richer in organic matter according to Eq. 1: *SE*(*D*) ≅ *D*(*p* + *q*), and that (b) the mean relative error of the estimation of carbon density in a sediment when using carbon fraction only to estimate bulk density through the mixing model will decrease as the sediment becomes richer in organic matter according to Eq. 2: *SE*(*D*) = *D*(*δ*/*δ_m_*)*p*.

These predictions were empirically tested using a Montecarlo simulation procedure, programmed as follows. (1) In a spreadsheet we generated 300 points representing sediments with random amounts of organic matter ranging from 0 to 100%, or carbon fraction ranging from 0 to 45%, assuming that organic matter content is 2.2 times the carbon fraction as discussed in Appendix S3. (2) Using the mixing model, we calculated for each point the bulk density of the sediment, to obtain two columns: (a) the “true” carbon content, and (b) the “true” bulk density. (3) We then calculated for each point a sample value of carbon and of gravimetric bulk density by adding to both values a normalized sampling error using as mean the true value, as standard deviation a fixed proportion (15%) of the true value, and a cumulative normal probability ranging randomly between zero and one. (4) Using the sample value for carbon fraction, we also calculated bulk density using the mixing model formula. (5) Multiplying the sample carbon fraction by the sample gravimetric bulk density, we obtained an estimate of carbon density (rescaled in our results to mg.cm^-3^) and plotted it against the true carbon content. (6) Finally, using the sample carbon fraction and the mixing model, we estimated carbon density and also plotted it against the true carbon content. This last simulation was done with parameter values for the mixing model of *δ_m_* = 1.75 g.cm^-3^ and *δ_p_* = 0.09 g.cm^-3^ (the mean of published values for the parameters). In order to assess the sensitivity of the carbon density estimation to the parameters, we repeated the Montecarlo simulation with *δ_m_* = 2.00 g.cm^-3^ and *δ_p_* = 0.10 g.cm^-3^ (the upper limit of reported values), and with *δ_m_* = 1.50 g.cm^-3^ and *δ_p_* = 0.08 g.cm^-3^ (the lower limit of reported values).

**References to Appendix S5**

Bohrnstedt, G. W., and A. S. Goldberger. 1969. “On the exact covariance of products of random variables.” *Journal* *of* *the* *American* *Statistical* *Association* **64**(328): 1439–1442. doi:10.1080/01621459.1969.10501069

Goodman, L. A. 1960. “On the exact variance of products.” *Journal* *of* *the* *American* *Statistical* *Association* **55**(292): 708–713. doi:10.1080/01621459
